# Ulnar Nerve Management in Distal Humerus Fracture Surgery: A Case of Developing Ulnar Neuropathy After Open Reduction and Internal Fixation

**DOI:** 10.7759/cureus.45477

**Published:** 2023-09-18

**Authors:** Muhammed Yusuf Afacan, Burak Ozturk, Mehmet Fatih Guven

**Affiliations:** 1 Department of Orthopedics and Traumatology, Istanbul University-Cerrahpasa, Cerrahpasa Medical Faculty, Istanbul, TUR

**Keywords:** iliac crest graft, nonunion, ulnar nerve transposition, distal humerus fracture, ulnar neuropathy

## Abstract

In this case report, we want to show how a patient who underwent surgery for a distal humerus fracture developed postoperative ulnar neuropathy symptoms, how nonunion persisted even at the ninth month of follow-up, and whether the nonunion was connected to the ulnar neuropathy that developed. Due to this, we used this case to explore ulnar nerve care and whether ulnar nerve transposition, manipulation, or decompression should be carried out during surgery on patients with distal humerus fractures. A 52-year-old man with a bi-columnar distal humerus fracture from a fall on his right elbow underwent open reduction and internal fixation at an external center one year before. Elbow restriction, discomfort, numbness, and weakness in the fourth and fifth digits of the right hand were all symptoms the patient experienced eight months following the surgery. We discovered the distal right humerus' nonunion during the radiological exams. It became apparent that the patient had no signs of ulnar neuropathy before the injury. In the eighth month following the injury, the patient had implant removal, open reduction internal fixation with autograft, and ulnar nerve transposition. We discovered during follow-up that the patient's ulnar neuropathy symptoms had subsided. The surgeon's familiarity with the procedure and command of the anatomy of the elbow has a role in managing the ulnar nerve in distal humerus fractures. We concluded that more study is required to determine the connection between the onset of ulnar neuropathy and nonunion while treating distal humerus fractures.

## Introduction

Distal humerus fractures account for 2%-6% of all fractures and 30% of elbow fractures in adults. Its incidence is higher in young men and older women. Most fractures in elderly patients are intra-articular fractures involving the medial and lateral columns. Treatment aims to provide anatomical reduction and stable fixation. After anatomical reduction and stable fixation, an early active joint range of motion should be started [1]. Complications such as nonunion, deformity, joint stiffness, and ulnar neuropathy may occur after surgery for distal humerus fractures. Stiffness, pain, and deformity are common complications following improper treatment and prolonged immobilization. The prognosis is worse for intra-articular multi-fragmented fractures, fractures accompanied by an open wound, and fractures with neurovascular deficit. The incidence of ulnar neuropathy after surgery for distal humerus fractures varies between 0% and 51%, with an average of 12% [2]. Most studies on the incidence of ulnar neuropathy after elbow trauma are retrospective, and it is unknown whether there was ulnar nerve dysfunction before the trauma [3]. The factor that causes ulnar neuropathy after trauma is not explicit. Manipulation of the nerve during surgery, heterotopic ossification, and scar tissue developing around the implant may cause ulnar neuropathy [4]. The frequency of neuropathy is independent of age, gender, fracture type, and whether the fracture is open or closed. The situation we want to show in this case report is the development of postoperative ulnar neuropathy findings in a patient who had surgery for a distal humerus fracture, the persistence of nonunion even at the ninth-month follow-up, and whether the ulnar neuropathy that developed is related to the nonunion. For this reason, we discussed whether ulnar nerve transposition, manipulation, or decompression should be performed during surgery on patients with distal humerus fractures and ulnar nerve management through this case.

## Case presentation

**Figure 1:**
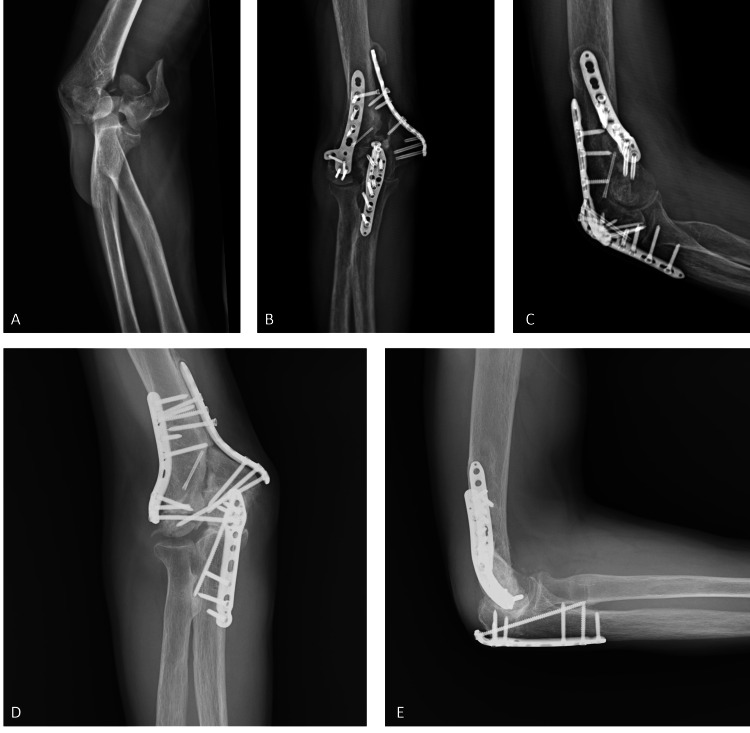
Radiographs of the patient after trauma, first surgery, and revision surgery. (A) Anteroposterior elbow radiograph of the patient after the trauma showing the bi-columnar distal humerus fracture. (B) Anteroposterior elbow radiograph of the patient before the revision surgery showing distal humerus nonunion and implant failure. (C) Lateral elbow radiograph of the patient before the revision surgery showing distal humerus nonunion and implant failure. (D) Anteroposterior elbow radiograph of the patient after the revision surgery. (E) Lateral elbow radiograph of the patient after the revision surgery.

A 52-year-old male patient applied to our clinic with elbow pain. He does not have a known comorbidity and does not have regular medication, has a smoking history of eight packs/year, and currently does not actively smoke. One year ago, open reduction and internal fixation were performed in an external center due to the development of a bi-columnar distal humerus fracture after falling on the right elbow. Eight months after the operation, the patient presented with limitation of movement in the right elbow, pain, numbness, and weakness in the fourth and fifth fingers of the right hand. In the examination, the distal humerus in the right elbow was painful with compression. In the right elbow joint range of motion examination, we observed 90 degrees of flexion and 30 degrees of extension limitation. There was no sign of instability in the elbow. There were signs of ulnar neuropathy in the right upper extremity. Radiological examinations revealed nonunion in the right humerus distal fracture (Figures 1A-1E). 

**Figure 2:**
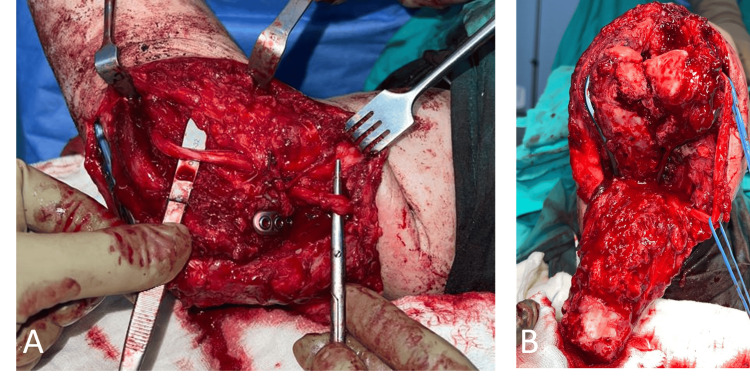
Intraoperative elbow photographs of the patient (A) after ulnar nerve transposition and (B) after autografting distal humerus with same-side iliac crest graft.

In the right upper extremity electromyography (EMG) examination performed before the operation after the patient had been admitted to our clinic, we determined findings consistent with partial axonal damage in the subacute-chronic period were detected distal to the lesion in the right ulnar nerve. The patient did not have ulnar neuropathy symptoms before the trauma. The patient underwent implant extraction, open reduction with iliac crest autograft, internal fixation, and ulnar nerve transposition in the 8th month after the trauma (Figures 2A, 2B).

We added Vitamin C, D, and B complex to his diet to support the healing of the ulnar neuropathy. After eight weeks of follow-up with a long arm splint, we started physical therapy and a rehabilitation program for both range of motion and ulnar neuropathy symptoms. We followed up with the patient until the sixth postoperative month. During follow-up, we detected regression of the patient's ulnar neuropathy symptoms.

## Discussion

When our patient was admitted to our clinic, nonunion of the distal humerus and signs of ulnar neuropathy were detected. We think that the loss of reduction and nonunion occurring after open reduction and internal fixation applied to our patient's distal humerus fracture may have occurred due to the incomplete immobilization of the elbow because of the ulnar neuropathy. We did not find any study on this subject in the literature. In a study comparing cases with internal fixation and total elbow arthroplasty for distal humerus fractures, the incidence of ulnar neuropathy after open reduction and internal fixation was 20% [3]. While the incidence of ulnar neuropathy in patients undergoing intraoperative decompression is 15.3%, the incidence of neuropathy in patients undergoing transposition is 23.5% [2]. The reason behind the higher incidence of ulnar neuropathy after transposition is that more dissection is required during transposition, resulting in trauma and devascularization [5]. In another study, no significant difference was detected between decompression and transposition [6]. In our clinic, we prefer anterior ulnar nerve transposition during internal fixation of distal humerus fractures, and the healing of the patient's fracture in the postoperative period and the regression of ulnar neuropathy findings show the success of our choice. In our patient, ulnar nerve transposition was not performed in his first operation for the distal humerus fracture. It has been a matter of debate whether the ulnar neuropathy findings that started after our patient's first operation were due to the contusion of the nerve after trauma or by fibrous tissue formed after the healing tissue in the surgical area. In some publications, transposing the ulnar nerve in a safe location while placing a plate during the operation is also responsible for ulnar neuropathy findings. However, some authors emphasized that in such a situation, only sensory deficit occurs, not accompanied by weakness [7]. Since our patient had an accompanying weakness, the neuropraxia findings are not related to this factor. Ulnar nerve transposition in surgery for distal humerus fractures has advantages such as preventing the nerve from bending by completely releasing it, preventing it from subluxation on the medial condyle, and reducing the potential for compression in scar tissue [8]. Some authors say that manipulating the ulnar nerve increases the risk of ulnar neuropathy [5,8]. In addition to the distal humerus fracture revision operation, the patient underwent anterior ulnar nerve transposition in our clinic, and our patient's neuropathy findings regressed after the surgery. As a result of our literature review, whether neuropathy findings would have occurred if ulnar nerve transposition had been performed in the first operation is a debatable issue.

## Conclusions

We expressed the differences in ulnar nerve management in the distal humerus fracture surgery. In the case of our patient, we discussed how the ulnar nerve should have been managed during his first operation and whether it would have changed the results. We wanted to emphasize the importance of ulnar nerve management in the surgical treatment of distal humerus fractures and the need to pay special attention to ulnar neuropathy in addition to the follow-up with the fracture healing process in the postoperative period. We think that ulnar nerve management in distal humerus fractures is related to the surgeon's familiarity with the technique and mastery of the elbow anatomy. We concluded that further research is necessary regarding the relationship between the development of ulnar neuropathy and nonunion in treating distal humerus fractures.
